# STAT3 suppression and β-cell ablation enhance α-to-β reprogramming mediated by Pdx1

**DOI:** 10.1038/s41598-022-25941-5

**Published:** 2022-12-10

**Authors:** Yuka Wakabayashi, Takeshi Miyatsuka, Masaki Miura, Miwa Himuro, Tomomi Taguchi, Hitoshi Iida, Yuya Nishida, Yoshio Fujitani, Hirotaka Watada

**Affiliations:** 1 Department of Metabolism and Endocrinology, Juntendo University Graduate School of Medicine, Tokyo, Japan; 2 Department of Endocrinology, Diabetes and Metabolism, Kitasato University School of Medicine, 1-15-1 Kitazato, Minami-Ku, Sagamihara, Kanagawa 252-0374 Japan; 3 Laboratory of Developmental Biology & Metabolism, Institute for Molecular & Cellular Regulation, Gunma University, Gunma, Japan; 4 Center for Identification of Diabetic Therapeutic Targets, Juntendo University Graduate School of Medicine, Tokyo, Japan; 5 Center for Therapeutic Innovations in Diabetes, Juntendo University Graduate School of Medicine, Tokyo, Japan

**Keywords:** Cell biology, Cell signalling, Insulin signalling, Biological techniques, Genetic engineering, Developmental biology, Differentiation

## Abstract

As diabetes results from the absolute or relative deficiency of insulin secretion from pancreatic β cells, possible methods to efficiently generate surrogate β cells have attracted a lot of efforts. To date, insulin-producing cells have been generated from various differentiated cell types in the pancreas, such as acinar cells and α cells, by inducing defined transcription factors, such as PDX1 and MAFA, yet it is still challenging as to how surrogate β cells can be efficiently generated for establishing future regenerative therapies for diabetes. In this study, we demonstrated that the exogenous expression of PDX1 activated STAT3 in α cells in vitro, and STAT3-null PDX1-expressing α cells in vivo resulted in efficient induction of α-to-β reprogramming, accompanied by the emergence of α-cell-derived insulin-producing cells with silenced glucagon expression. Whereas β-cell ablation by alloxan administration significantly increased the number of α-cell-derived insulin-producing cells by PDX1, STAT3 suppression resulted in no further increase in β-cell neogenesis after β-cell ablation. Thus, STAT3 modulation and β-cell ablation nonadditively enhance α-to-β reprogramming induced by PDX1, which may lead to the establishment of cell therapies for curing diabetes.

## Introduction

As diabetes mellitus results from the absolute or relative deficiency of insulin secretion from pancreatic β cells^1^, the generation of insulin-producing cells has been a target for the cure of diabetes. To reach this ultimate goal, many attempts have been made to generate surrogate β cells from human embryonic stem cells^2,3^ or other differentiated cell types, such as pancreatic acinar cells, ductal cells, and glucagon-expressing α cells^4–6^. We consider that α cells are a prime candidate for reprogramming into β cells, because they are developmentally closely related to β cells. Notably, mouse islet α cells transdifferentiate into β cells under conditions of extreme β-cell loss, which is a condition similar to type 1 diabetes^7,8^. We and others have previously shown that the ectopic expression of β-cell specific transcription factors, pancreatic and duodenal homeobox 1 (PDX1) and musculoaponeurotic fibrosarcoma oncogene family A (MAFA), converts both embryonic or adult α cells into insulin-producing cells in mice^9,10^. Likewise, human islet α cells can be reprogrammed into insulin-positive cells by adenovirus-mediated expression of PDX1 and MAFA^11^. Thus, while both PDX1 and MAFA have been demonstrated to play essential roles in α-to-β reprogramming, identifying efficient methods to generate surrogate β cells, which will lead to the establishment of regenerative therapies for people with diabetes, still remains a challenge.

Modulating cellular plasticity may be the key to improve reprogramming efficiency into the β-cell lineage. STAT3 has been demonstrated to play a role in regulating cellular plasticity in various cell types, such as pluripotent stem cells^12,13^ and hematopoietic cells^14^. In addition, we previously demonstrated that STAT3 regulates cellular identities in pancreatic acinar cells^6,15^. Notably, activating mutations in human *STAT3* have been reported to cause neonatal diabetes accompanied by β-cell failure^16,17^. Thus, as proper STAT3 activity appears to be essential for determing the cellular identities of pancreatic cells as well as other cells types, we hypothesized that modifying STAT3 signaling may contribute to the reprogramming efficiency into insulin-producing cells, not only for acinar cells but also for α cells. To address this issue, we developed experimental models to investigate the role of STAT3 in α-to-β reprogramming induced by Pdx1, and demonstrated that the suppression of STAT3 signaling enhances the reprogramming efficiency into β cells.

Another key to enhance cellular reprogramming into β cells is the extreme ablation of β cells, as previously demonstrated^7,8^. To further investigate the effects of β-cell ablation on α-to-β reprogramming, we induced the ectopic expression of PDX1 in α cells combined with β-cell ablation by injecting alloxan, demonstrating that extreme β-cell loss robustly enhances α-to-β reprogramming induced by Pdx1, although there was no additive effect by Stat3 inhibition.

## Results

### Ectopic expression of Pdx1 induced STAT3 activation in α cells

**Figure 1:**
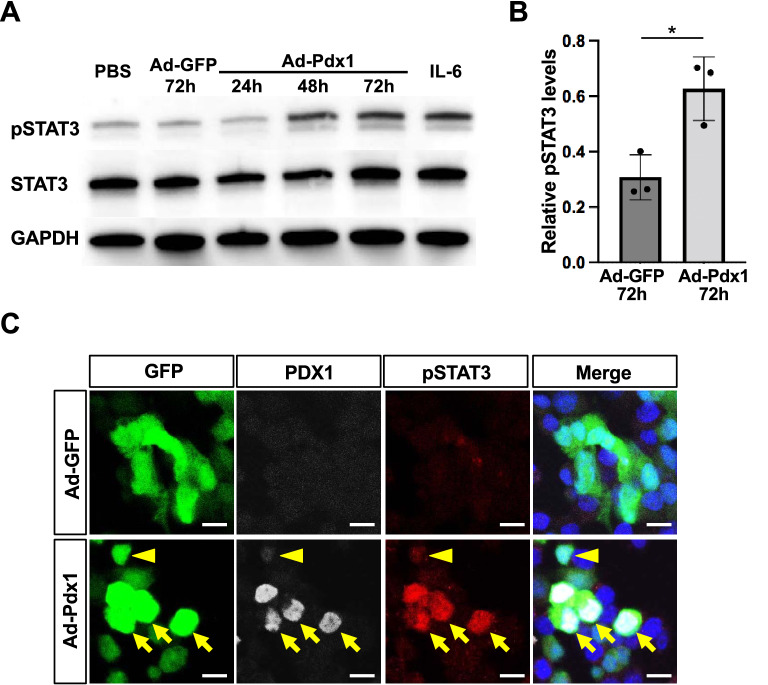
Ectopic expression of Pdx1 induces STAT3 activation in α cells. (**A**) αTC1cells were infected with Pdx1-expressing adenovirus (Ad-Pdx1) or a control adenovirus expressing GFP(Ad-GFP). Protein levels of phosphorylated STAT3 (pSTAT3), total STAT3, and GAPDH were assessed by Western blotting. IL-6-treated cells were used as a positive control. (**B**) Quantification of the Western blot shown in (**A**). The expression levels of pSTAT3 were normalized to those of total STAT3. *p < 0.05 (n = 3 for each group) (**C**) Immunocytochemical staining for Pdx1 (white) and phospho-STAT3 (pSTAT3, red) was performed in αTC1 cells 72 h after infection with a control adenovirus (Ad-GFP) or an adenovirus expressing Pdx1(Ad-Pdx1). STAT3 was activated in αTC1 cells with high expression of PDX1 (arrows), whereas STAT3 was not activated in cells with weak expression of PDX1 (arrowhead). Scale bars, 20 μm. The original blots/gels are presented in Fig. S10.

STAT3 has been shown to be activated in pancreatic acinar cells that ectopically express Pdx1^6,15^. To investigate whether STAT3 is activated in pancreatic α cells as well as in acinar cells, an adenoviral vector expressing Pdx1 (Ad-Pdx1) was infected into αTC1 cells, a mouse glucagonoma cell line. Immunoblotting for phosphorylated STAT3 (pSTAT3) at Tyr705 demonstrated a significant increase in pSTAT3 levels in αTC1 cells infected with Ad-Pdx1, compared with control αTC1 cells infected with a green-fluorescent protein (GFP)-expressing adenovirus (Ad-GFP) 72 h after adenoviral infection (Fig. 1A,B). In addition, immunocytochemical staining clearly detected pSTAT3 protein in Ad-Pdx1-infected αTC1 cells, with high expression of PDX1, whereas few nuclei were positive for pSTAT3 in cells with weak expression of PDX1, and in control cells infected with Ad-GFP (Fig. 1C). When αTC1 cells were infected with an adenoviral vector expressing Mafa (Ad-Mafa), another β-cell-specific transcription factor, the exogenous expression of Mafa did not activate STAT3 (Fig. S1). This is in contrast to our previous findings in mPAC cells, which exhibit pancreatic progenitor-like characteristics^6^. Taken together, these findings suggest that Pdx1 activates STAT3 in α cells as well as in acinar cells.

### STAT3 deletion enhances α-to-β reprogramming induced by Pdx1

**Figure 2:**
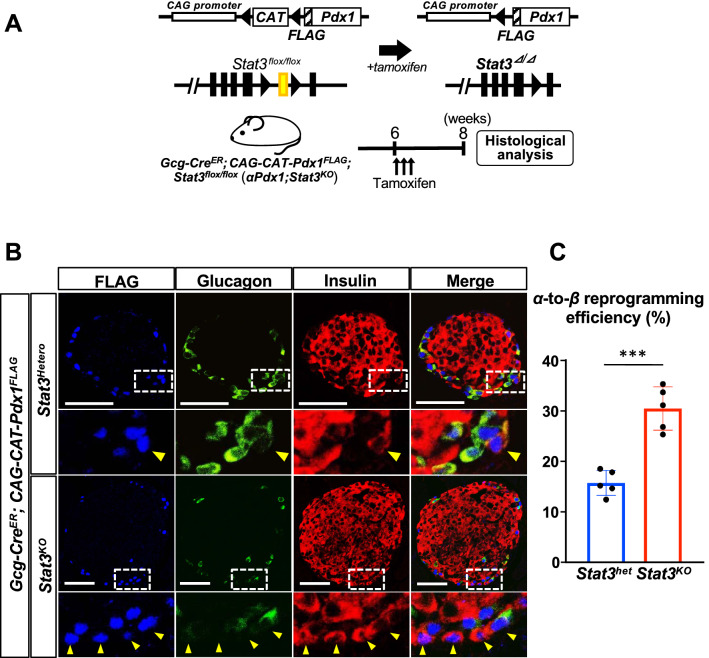
STAT3 deletion enhances α-to-β reprogramming induced by Pdx1. (**A**) Schematic representation of the transgenes and their Cre-mediated recombination in *Gcg-Cre*^*ER*^*; CAG-CAT-Pdx1*^*FLAG*^*; Stat3*^*flox/flow*^ (*αPdx1; Stat3*^*KO*^) mice. Before recombination, the transcription of Pdx1 is blocked by the floxed STOP cassette. When the mice are treated with tamoxifen, the floxed sequence is removed by Cre recombinase, and the CAG promoter activates the expression of Pdx1. Likewise, the loxP-flanked (floxed) Stat3 gene is removed. (**B**) Immunostaining for FLAG-tagged Pdx1 (blue), glucagon (green) and insulin (red) in the pancreas of *αPdx1; Stat3*^*KO*^ mice 2 weeks after tamoxifen administration. The arrowheads show FLAG-tag positive cells which express insulin. Scale bars, 50 μm. Magnified images of the dotted square regions are shown below each image. (**C**) The percentage of reprogrammed-β cells among FLAG-tag positive cells. ***p < 0.005 (n = 5 in each group).

Pdx1 has been shown to change α-cell fate into the β-cell lineage both in vitro and in vivo^18,19^. On the other hand, we previously demonstrated that Stat3 plays a role in modulating cell fates orchestrated by Pdx1^6,15^. These findings led us to hypothesize that Stat3 plays a role in modulating the α-to-β reprogramming induced by Pdx1. When Stat3 signaling was suppressed in Ad-Pdx1-infected αTC1 cells using an adenovirus expressing a dominant-negative form of STAT3 (Ad-Stat3-DN), there was no difference in the expression levels of *Ins1* and *Ins2* mRNAs between cells with and without Ad-Stat3-DN (Fig. S2). Next, to investigate our hypothesis in vivo, *Gcg-Cre*^*ER*^ mice, in which Cre-mediated recombination is induced in α cells, were sequentially crossed with *CAG-CAT-Pdx1*^*FLAG*^ mice and floxed *Stat3* mice to generate *Gcg-Cre*^*ER*^; *CAG-CAT-Pdx1*^*FLAG*^*; Stat3*^*flox/flox*^ (*αPdx1; Stat3*^*KO*^) mice (Fig. 2A). Six-week-old *αPdx1; Stat3*^*KO*^ mice were subcutaneously injected with tamoxifen to induce the ectopic expression of Pdx1, together with STAT3 deficiency, specifically in α cells. Double immunostaining against FLAG-tag and pSTAT3 demonstrated that 7.8% ± 2.3% of FLAG-tag-positive cells expressed pSTAT3 in *αPdx1; Stat3*^*Hetero*^ mice 7 days after tamoxifen administration, whereas few FLAG-tag-positive cells (0.6% ± 0.3%) were positive for pSTAT3 in *αPdx1; Stat3*^*KO*^ mice (Fig. S2). The substantial decrease in the number of pSTAT3/FLAG-tag double-positive cells was also observed 1 and 3 days after tamoxifen administration in *αPdx1; Stat3*^*KO*^ mice, suggesting that *Stat3* was inactivated after Cre-mediated recombination, as originally designed (Fig. S3). As shown in the Fig. 2B, a substantial number of α cells expressed exogenous Pdx1 detected by anti-FLAG antibody. FLAG-tag-labeled insulin-producing cells were also detected, which are α-cell-derived insulin-producing cells. We calculated the α-to-β reprogramming efficiency as the number of FLAG-tag/insulin double-positive cells divided by the total number of FLAG-tagged cells (Fig. 2C), which demonstrated that Stat3 deletion resulted in a significantly larger number of α-cell-derived insulin-producing cells in the islets of *αPdx1; Stat3*^*KO*^ mice than in the islets of *Gcg-Cre*^*ER*^*; CAG-CAT-Pdx1*^*FLAG*^*; Stat3*^*flox/*+^ (*αPdx1; Stat3*^*Hetero*^) mice (30.9% ± 1.9% vs. 16.0% ± 1.1%) 2 weeks after Cre-mediated recombination. Evaluation at shorter time periods after Cre-mediated recombination resulted in no significant differences in α-to-β reprogramming efficiency between the groups (Fig. S4). STAT3 deletion itself, without the ectopic expression of Pdx1, did not increase α-to-β reprogramming efficiency (Fig. S5). These findings demonstrate that Stat3 deletion enhances α-to-β reprogramming in vivo induced by Pdx1.

### STAT3 deletion together with Pdx1 expression induces α-to-β reprogramming with silenced glucagon expression

**Figure 3:**
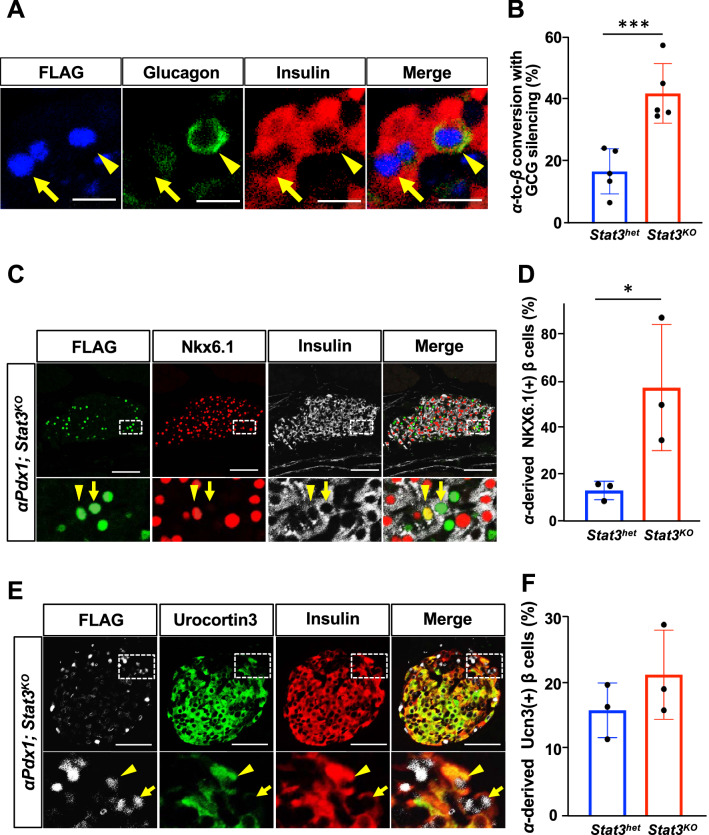
The Characteristics of α-cell-derived insulin-producing cells. (**A**) Representative images of two types of insulin-producing cells derived from α cells. Immunostaining for FLAG-tagged Pdx1 (blue), glucagon (green), and insulin (red) was performed on pancreas sections of *αPdx1; Stat3*^*KO*^ mice. A FLAG-tag/insulin double positive cell that is negative for glucagon staining (arrow), which denotes Gcg-silenced α-to-β conversion, and a FLAG-tag positive cell expressing both insulin and glucagon (arrowhead), which denotes α-to-bihormonal transition are shown. Scale bars, 10 μm. (**B**) The percentage of α-cell-derived insulin-expressing cells that do not express glucagon among total α-cell-derived β cells. ***p < 0.005 (n = 5 in each group). (**C**) Immunostaining for FLAG-tagged Pdx1 (green), Nkx6.1 (red) and insulin (white) in pancreas sections of *αPdx1; Stat3*^*KO*^ mice 2 weeks after tamoxifen administration. The arrowhead indicates a FLAG-tag-positive cell that expresses both Nkx6.1 and insulin. The arrow indicates FLAG-tag/insulin double positive cells that lack Nkx6.1. Scale bars, 50 μm. Magnified images of the dotted square regions are shown below each image. (**D**) Percentage of Nkx6.1-positive cells among FLAG-tag/insulin double-positive cells (α-cell-derived β cells). Pancreata of *Stat3*-knockout mice have a significantly higher number of α-cell-derived Nkx6.1-expressing β cells than pancreata of control mice. *p < 0.05 (n = 3 in each group). (**E**) Immunostaining for FLAG-tagged Pdx1 (white), UCN3 (green) and insulin (red) in pancreas sections of *αPdx1; Stat3*^*KO*^ mice 2 weeks after tamoxifen administration. The arrowhead indicates a FLAG-tag positive cell that expresses both urocortin3 and insulin. The arrow indicates a FLAG-tag/insulin double-positive cell that lacks urocortin3. Magnified images of the dotted square regions are shown below each image. (**F**) The percentage of urocortin3-positive cells among FLAG-tag/insulin double-positive cells (α-derived β cells, n = 3 in each group).

We next performed triple immunostaining against FLAG-tag, insulin, and glucagon, and found that there were two types of FLAG-tagged, i.e., α-cell-derived, insulin-expressing cells; some cells expressed both insulin and glucagon, which denotes α-to-bihormonal conversion, whereas other cells expressed FLAG-tagged PDX1 and insulin without expressing glucagon, which denotes glucagon-silenced α-to-β conversion (Fig. 3A). The number of α-cell-derived insulin-expressing cells, which were negative for glucagon, was significantly larger in the pancreata of *αPdx1; Stat3*^*KO*^ mice than in the pancreata of *αPdx1; Stat3*^*Hetero*^ mice (41.9% vs. 16.8%, Fig. 3B). As insulin/glucagon double-positive cells are thought to be immature cells in the developing pancreas^20^, this finding suggests that Stat3 suppression enhanced more advanced α-to-β conversion induced by Pdx1.

### Stat3 deletion modifies the characteristics of α-cell-derived β cells

To further investigate the characteristics of α-cell-derived insulin-expressing cells, immunostaining against NKX6.1 and urocortin 3 (UCN3), which are highly expressed in endogenous β cells^21,22^, was performed. Although the number of α-cell-derived Nkx6.1-expressing cells at 1, 3, and 7 days after tamoxifen administration was comparable between *Stat3-*heterozygous and *Stat3*-deficient mice, it was significantly increased 2 weeks after tamoxifen administration in *Stat3*-deficient mice compared with *Stat3-*heterozygous mice (57.4% ± 15.7% vs. 13.1% ± 2.3%, respectively, Fig. 3C,D, and Fig. S6). In contrast, there was no significant difference in the number of UCN3-expressing cells in both groups (Fig. 3E,F, and Fig. S7). These findings suggest that Stat3 deletion together with the ectopic expression of Pdx1 may endow α cells with some β-cell characteristics to some extent, and further steps are necessary to induce the cellular reprogramming of α cells into more fully differentiated β cells that are indistinguishable from endogenous β cells.

### Alloxan-induced β-cell ablation promotes α-to-β reprogramming induced by Pdx1

**Figure 4:**
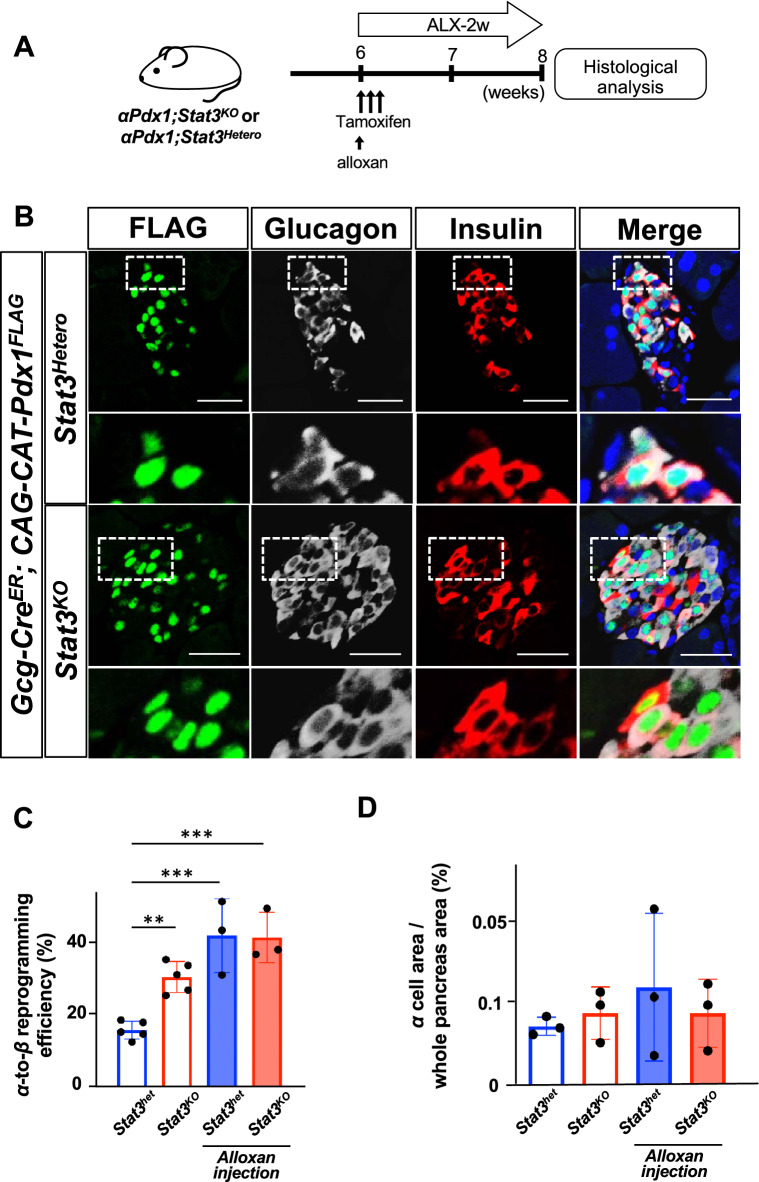
Alloxan-induced β-cell ablation promotes α-to-β reprogramming by Pdx1. (**A**) Experimental design of β-cell ablation and induction of α-to-β reprogramming. At 6 weeks of age, alloxan was administered into the *αPdx1; Stat3*^*KO*^ mice. For the induction of Cre-mediated recombination, mice were subcutaneously injected with 4 mg of tamoxifen 3 times over a 1-week period. (**B**) Immunostaining for FLAG-tagged Pdx1 (green), glucagon (white) and insulin (red) in pancreas sections of *αPdx1; Stat3*^*KO*^ mice 2 weeks after alloxan and tamoxifen administration. Scale bars, 50 μm. Magnified images of the dotted square regions are shown below each image. (**C**) The percentage of reprogrammed-β cells among FLAG-tag positive cells. **p < 0.01, ***p < 0.005 (n = 3–5 in each group) (**D**) Percentage of α cell area among whole pancreas area (n = 3 in each group).

It has been reported that extreme β-cell ablation induces α-to-β conversion in mice^7,8^. To investigate whether β-cell ablation affects the reprogramming efficiency and/or the characteristics of reprogrammed β cells in our experimental model, we induced β-cell ablation by injecting alloxan (ALX) into *αPdx1; Stat3*^*KO*^ mice and control *αPdx1; Stat3*^*Hetero*^ mice (Fig. 4A and Fig. S8). The reprogramming efficiency induced by Pdx1 was significantly increased after β-cell ablation in both mice with heterozygous and homozygous mutations of the *Stat3* gene, compared with normoglycemic *αPdx1; Stat3*^*Hetero*^ mice without ALX injection (Fig. 4B,C). In contrast, there was no difference in reprogramming efficiencies between *Stat3*^*KO*^ and *Stat3*^*Hetero*^ mice after β-cell ablation (42.6% ± 6.0% vs. 42.1% ± 4.1%, Fig. 4C). There was no significant difference in α-cell mass between the groups, with or without ALX injection (Fig. 4D), showing that neither the ectopic expression of Pdx1 nor β-cell ablation by ALX substantially affected the homeostasis of α-cell volume. These findings suggest that STAT3 deletion and β-cell ablation nonadditively enhance the α-to-β reprogramming induced by Pdx1.

### Reduced α-to-β reprogramming efficiency in aged mice

To investigate whether aging affects α-to-β reprogramming efficiency in mice, the number of α-cell-derived insulin-producing cells was quantified in *Stat3*^*KO*^ and *Stat3*^*Hetero*^ mice at the age of 28 weeks or older. As shown in Fig. S9, there were fewer reprogrammed β cells in the aged mice than in 8-week-old mice (Fig. 2). In addition, STAT3 deletion did not significantly enhance α-to-β reprogramming.

## Discussion

Whereas the transcription factor Pdx1 has been demonstrated to endow pancreatic cells with some β-cell characteristics both in vitro and in vivo^18,19^, the generation of fully functional β cells from α cells remains a challenge. We previously reported that the suppression of STAT3 signaling enhanced the cellular reprogramming of acinar cells into β cells, and the present study further demonstrated the significance of Stat3 signaling in α-to-β reprogramming in mice.

STAT3 has been shown to play various roles in cell differentiation and proliferation, and in maintaining pluripotency in iPS/ES cells^12,13^, cancer stem cells^23^, and hematopoietic stem cells^14^. In addition, the crucial role of STAT3 signaling in the regulation of pancreatic cellular plasticity has been demonstrated in previous in vivo studies, including ours^6,15,24^. Furthermore, activating mutations in STAT3 have been reported to cause neonatal diabetes in humans^16,17^. Based on these previous findings together with our present study, STAT3 signaling appears to play an essential role in maintaining the cellular identity of pancreatic α cells as well as acinar cells, and the suppression of STAT3 can enhance cellular reprogramming into β cells, orchestrated by the ectopic expression of PDX1.

Previous studies have demonstrated that the combined ectopic expression of Pdx1 and Mafa efficiently induces the cellular reprogramming of α cells into insulin-producing cells in mice and humans^9–11^. Although the transgenic expression of Pdx1 alone in the α-cell lineage without β-cell ablation was shown to have no or little effect on β-cell genesis^9,25^, the exogenous expression of Pdx1 alone successfully induced α-to-β reprogramming in our present study. As the studies by Matsuoka et al. and Cigliola et al. both used the CAG-CAT-Pdx1^FLAG^ mice that we previously generated^26^, the expression levels of PDX1 in the mice are expected to be the same after Cre-mediated recombination in our study and these previous studies. One of the obvious differences is that tamoxifen-inducible *Gcg-Cre*^*ER*^ mice were used to induce Cre-mediated recombination in the present study, whereas *Gcg-Cre* or *Gcg-rtTA; TetO-Cre* mice were used in other studies. Tamoxifen treatment may have a beneficial effect in enhancing α-to-β reprogramming. In addition, even heterozygous deletion of the *Stat3* gene may affect α-cell plasticity in *αPdx1; Stat3*^*Hetero*^ mice. Interestingly, another previous study showed that the ectopic expression of Pdx1 alone into sorted human α cells using adenovirus induced insulin gene expression in α cells^11^. Thus, the ectopic expression of Pdx1 alone is likely to endow α cells with β-cell characteristics under some specific experimental conditions.

Not only STAT3 deletion but also β-cell ablation enhanced the α-to-β reprogramming induced by Pdx1. As there was no additive effect between Stat3 inhibition and β-cell ablation, insulin insufficiency by β-cell ablation may stimulate the same downstream pathways as Stat3 inhibition. Another possibility is that Stat3 inhibition may induce α-to-β reprogramming in coordination with insulin signaling, and may have little effect under insulin insufficiency. Further studies are needed to clarify the underlying molecular mechanisms involved, to maximize the reprogramming efficiency into β cells, which is expected to lead to the establishment of future cell therapies for the cure of diabetes.

## Methods

### Cell culture

The mouse pancreatic α-cell line αTC1 (clone 6) was purchased from American Type Culture Collection (Manassas, VA, USA). The cells were cultured in DMEM with 10% fetal bovine serum and incubated at 37 °C in an atmosphere of 5% CO_2_ in air.

### Preparation of adenoviruses.

Recombinant adenoviruses expressing Pdx1 (Ad-Pdx1) were generated as described previously^27^. As each adenovirus used in this study carries GFP, adenovirus-infected cells are labeled with green fluorescence. An adenovirus expressing only GFP was used as a control (Ad-GFP).

### Western blotting

Whole-cell protein extracts were isolated using RIPA lysis buffer (Thermo Scientific, Rockford, IL, USA) containing protease inhibitor cocktail (Thermo Scientific). Ten micrograms of total proteins were loaded and fractionated by SDS-PAGE, transferred to nitrocellulose membranes (Merck Millipore, Darmstadt, Germany), and probed with primary antibodies against pSTAT3, total STAT3 (rabbit, 1:1000; Cell Signaling Technology, Danvers, MA, USA), and GAPDH (rabbit, 1:1000; Cell Signaling Technology). Immunoreactivity was visualized using SuperSignal West Extended Duration Substrate (Thermo Fisher Scientific, Waltham, MA, USA) according to the manufacturer’s instructions. The protein extracts from αTC1 cells treated with IL-6 were used as a positive control. The expression levels of pSTAT3 were normalized to those of total STAT3.

### Mice

*CAG-CAT-Pdx1*^*FLAG*^, *Gcg-Cre*^*ER*^, *ROSA26*^*mTmG*^, and *floxed-Stat3* mice were generated as previously described^6,26,28–31^. *Gcg-Cre*^*ER*^ mice, which express tamoxifen-activated Cre recombinase in α cells, were crossed with *Pdx1*^*FLAG*^ mice to induce α-to-β reprogramming. Floxed Stat3 mice were repeatedly crossed with *Gcg-Cre*^*ER*^*; Pdx1*^*FLAG*^ mice to generate *Gcg-Cre*^*ER*^*; CAG-CAT-Pdx1*^*FLAG*^*; Stat3*^*KO*^ mice. *Gcg-Cre*^*ER*^*; CAG-CAT-Pdx1*^*FLAG*^*; Stat3*^*KO*^ or control *Gcg-Cre*^*ER*^*; CAG-CAT-Pdx1*^*FLAG*^*; Stat3*^*Hetero*^ mice are viable, fertile, and indistinguishable from their wild-type (WT) littermates with respect to weight, blood glucose, and glucose tolerance. To induce Cre-mediated recombination, tamoxifen (Sigma Aldrich, St. Louis, MO, USA) was dissolved in corn oil at 20 mg/mL and injected subcutaneously at 2 mg/10 g body weight, 3 times over a 1-week period. The mice were euthanized at 1, 3, 7, and 14 days after tamoxifen administration.

At 6 weeks of age, ALX (Sigma Aldrich) was administered into the mice as a single intravenous injection at a dose of 100 mg/kg body weight through the tail vein. For induction of Cre-mediated recombination, tamoxifen was subcutaneously injected 3 times over a 1-week period.

Mice were maintained on a 12-h light/dark cycle in a controlled atmosphere and fed standard rodent food. The study protocol was reviewed and approved by the Animal Care and Use Committee of Juntendo University. All methods were carried out in accordance with relevant guidelines and regulations, and are reported in accordance with ARRIVE guidelines.

### Histological analysis

Tissues were harvested and fixed in 4% paraformaldehyde in PBS, and embedded in paraffin, for subsequent sectioning (5-μm thickness). For immunofluorescence analysis of paraffin-embedded tissues, sections were deparaffinized in xylene and dehydrated in graded ethanol before heat-induced epitope retrieval in a microwave oven (95 °C for 15 min in 10 mM citrate buffer). Slides were then blocked in 1% horse serum (Vector Laboratries, Burlingame, CA, USA) and incubated overnight in primary antibody. The primary antibodies used in this study were the following: rabbit anti-pSTAT3 (1:100; Cell Signaling Technology), guinea pig anti-insulin (1:5; Dako, Carpinteria, CA, USA), rat anti-insulin (1:200; R&D Systems, Minneapolis, MN, USA), rabbit anti-glucagon (1:1000; Dako), guinea pig anti-glucagon (1:1000; Takara Bio, Shiga, Japan), mouse anti-FLAG (1:200; TransGenic, Fukuoka, Japan). Then, slides were incubated with secondary antibody for 30 min at room temperature. The secondary antibodies used were Alexa Fluor 633-conjugated anti-rat IgG, Alexa Fluor 633-conjugated anti-guinea pig IgG, Alexa Fluor 568-conjugated anti-rat IgG, Alexa Fluor 555-conjugated anti-rabbit IgG, Alexa Fluor 555-conjugated anti-guinea pig IgG, Alexa Fluor 488-conjugated anti-guinea pig IgG, Alexa Fluor 488-conjugated anti-rabbit IgG, Alexa Fluor 488-conjugated anti-rat IgG (all at 1:200; Invitrogen, Carlsbad, CA, USA). Cell nuclei were stained with 4,6-diamidino-2-phenylindole (DAPI; Vector Laboratories). After washing in PBS, slides were mounted in Vectashield mounting medium (Vector Laboratories). Images were captured with a laser scanning confocal microscope (Zeiss LSM 780).

### Statistical analyses

Statistical analyses were performed using GraphPad Prism software (GraphPad Software, La Jolla, CA, USA). Comparisons of two samples were performed by the unpaired two-tailed *t*-tests. Multiple groups were analyzed by one-way ANOVA by a multiple comparison test. A *p*-value of less than 0.05 was considered to indicate a statistically significant difference between two groups. All data are presented as the mean ± SE.

## Supplementary Information

Supplementary Information.

## Data Availability

The datasets generated and/or analyzed during the current study are available from the corresponding author on request.
