# Supplementary material for: STAT3 suppression and β-cell ablation enhance α-to-β reprogramming mediated by Pdx1
Source: Sci Rep. 2022 Dec 10;12:21419. doi: 10.1038/s41598-022-25941-5 (PMC9741642; doi:10.1038/s41598-022-25941-5)
Supplement: Supplementary file 1 — Supplementary Information. [file 41598_2022_25941_MOESM1_ESM.pdf]

Figure S1

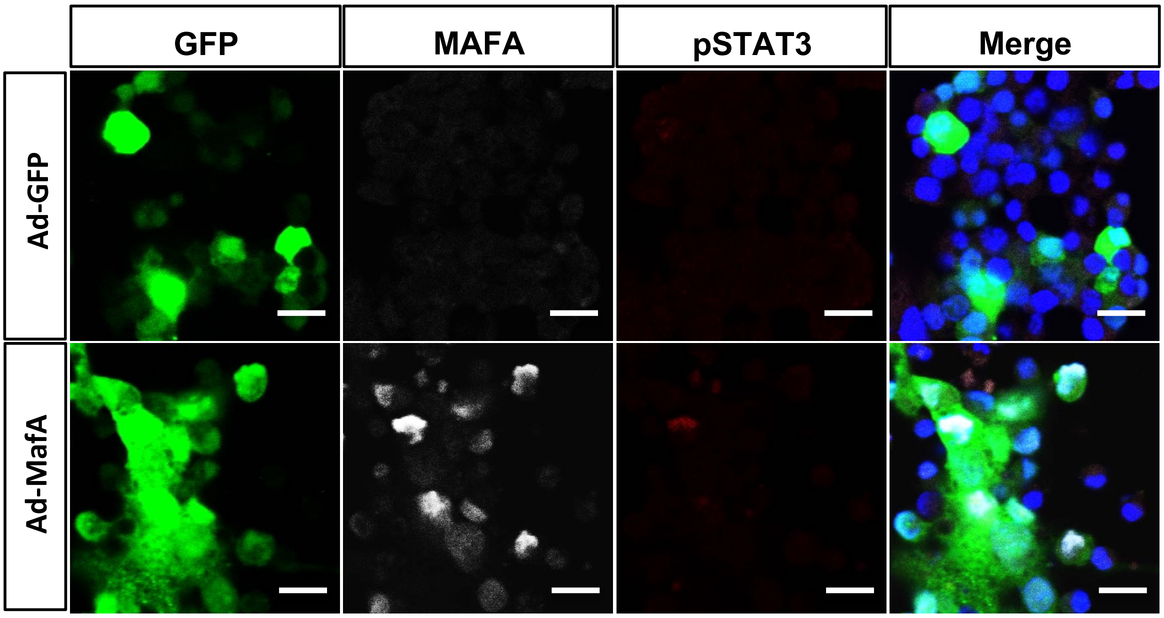

Figure S1. No activation of STAT3 in αTC1 cells treated with a Mafa-expressing adenovirus. Immunostaining against MafA (white) and phospho-STAT3 (pSTAT3, red) was performed in αTC1 cells 48 hours after infection with a control adenovirus (Ad-GFP) or an adenovirus expressing MafA (Ad-MafA). Scale bars, 20 µm.

Figure S2

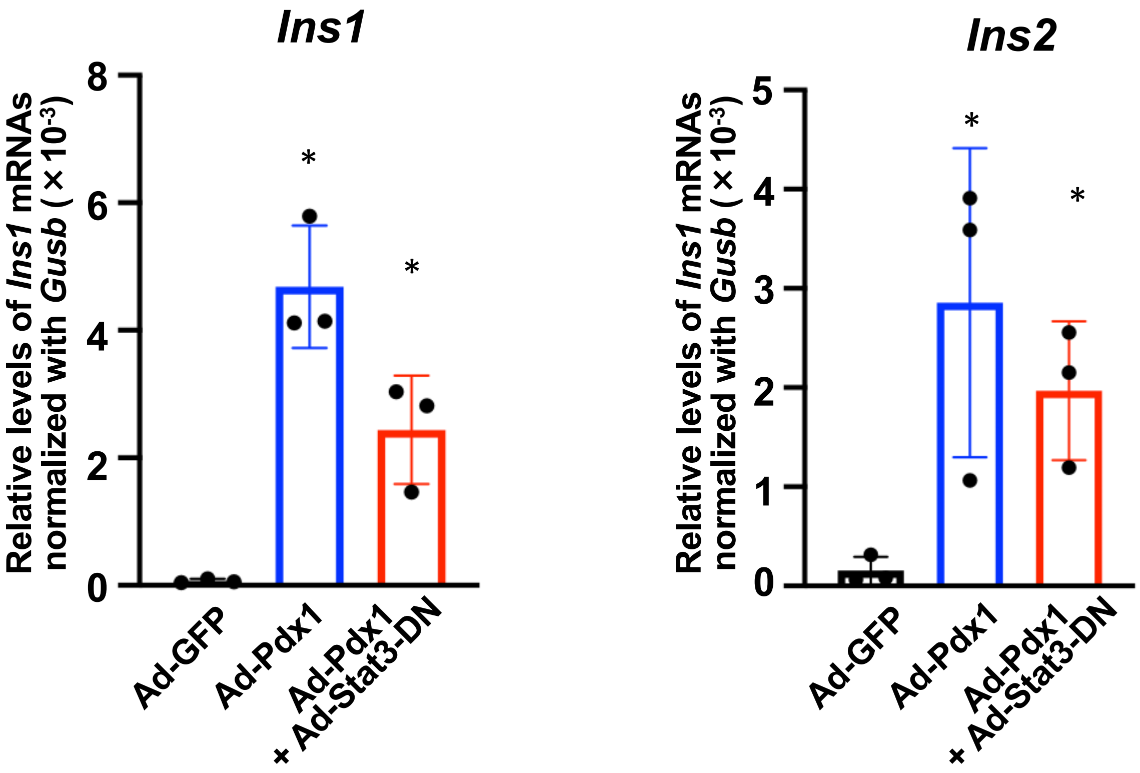

Figure S2. *Ins1* and *Ins2* expression in αTC1 cells infected with Ad-STAT3DN.
The αTC1 cells were infected with Pdx1-expressing adenovirus (Ad-Pdx1), together with an adenovirus expressing a dominant-negative form of STAT3 (Ad-STAT3DN), and quantitative PCR was performed 3 days after the adenoviral infection. Relative expression levels of *Ins1* and Ins2 normalized with *Gusb* were quantified (n = 3 in each group). *, $p < 0.05$ versus Ad-GFP.

Figure S3

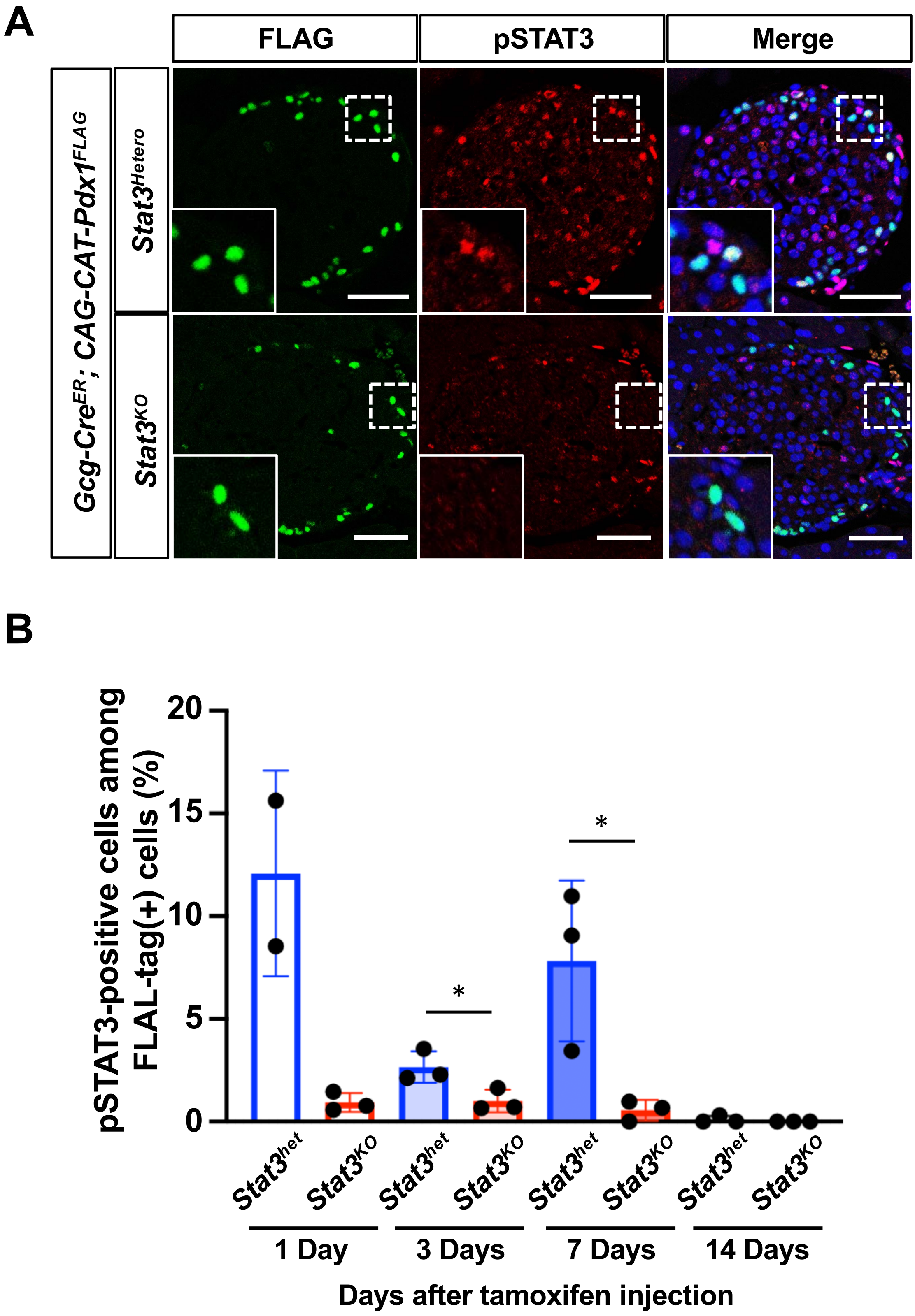

Figure S3. Induction of STAT3-deficiency in *αPdx1; $Stat3^{KO}$* mice.
(A) Immunostaining against FLAG-tagged Pdx1and pStat3 was performed in *αPdx1; $Stat3^{Hetero}$* and *αPdx1; $Stat3^{KO}$* mice 7 days after tamoxifen administration. Magnified images of the dotted square are shown in the bottom left of each image. (B) Percentage of pSTAT3-positive cells among FLAG-tag positive cells. *, $p < 0.05$ (n=2 in *αPdx1; $Stat3^{Hetero}$* mice sacrificed 1 day after tamoxifen injection, n=3 in other groups).

Figure S4

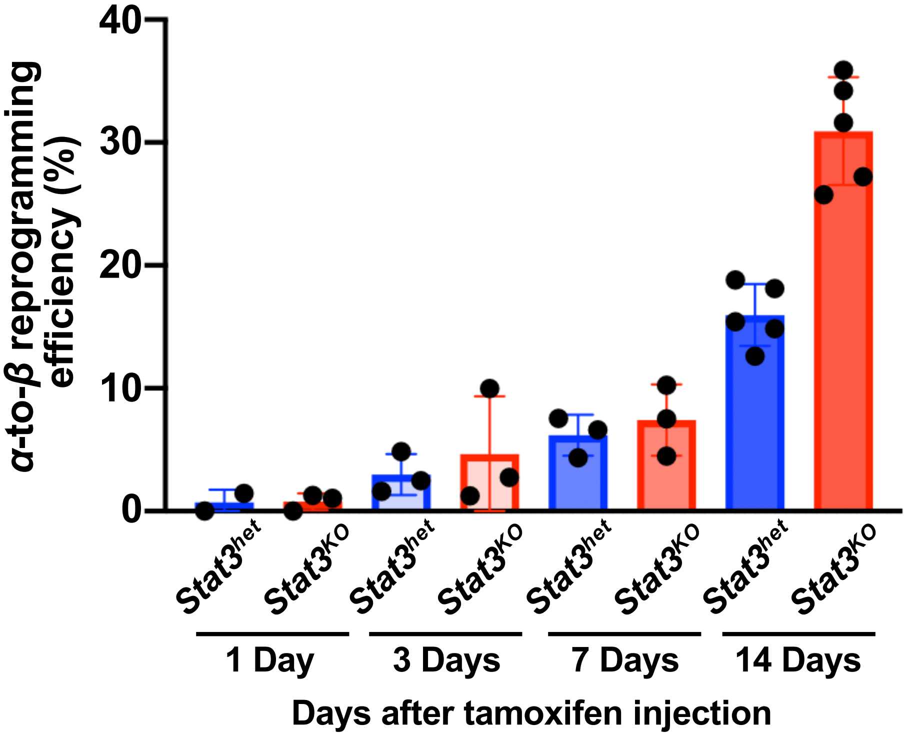

Figure S4. Temporal increase in α-to-β reprogramming after Cre-mediated recombination. The percentage of reprogrammed-β cells among FLAG-tag-positive cells was calculated in *αPdx1; Stat3*^{Hetero} and *αPdx1; Stat3*^{KO} mice 3, 7, and 14 days after tamoxifen administration (n = 3–5 in each group).

Figure S5

A

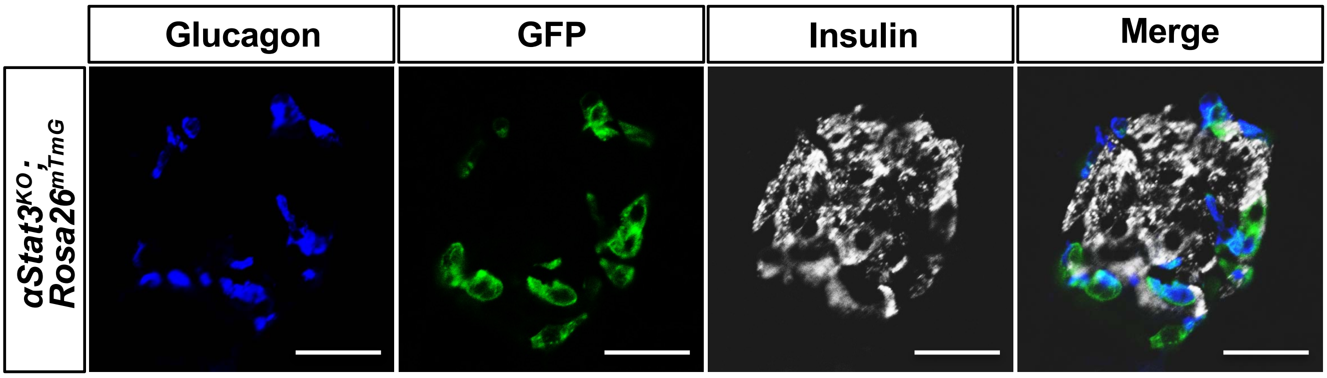

B

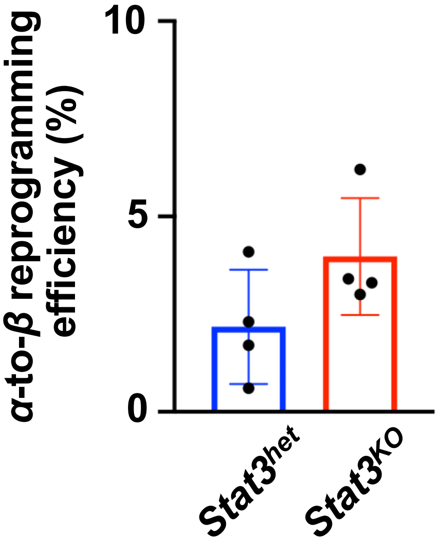

Figure S5. STAT3-deficiency without ectopic expression of PDX1 did not enhance α-to-β reprogramming.
(A) Immunostaining against insulin (white) and glucagon (blue) was performed in the pancreas of *Gcg-Cre^{ER}; Stat3$^{flox/flox}$; Rosa26^{mTmG}* mice. Scale bars, 50 μm. (B) The percentage of insulin-positive cells among GFP-expressing cells in α-cell lineage was calculated in *Gcg-Cre^{ER}; Stat3$^{flox/+}$; Rosa^{mTmG}* and *Gcg-Cre^{ER}; Stat3$^{flox/flox}$; Rosa^{mTmG}* mice (n=4 in each group).

Figure S6

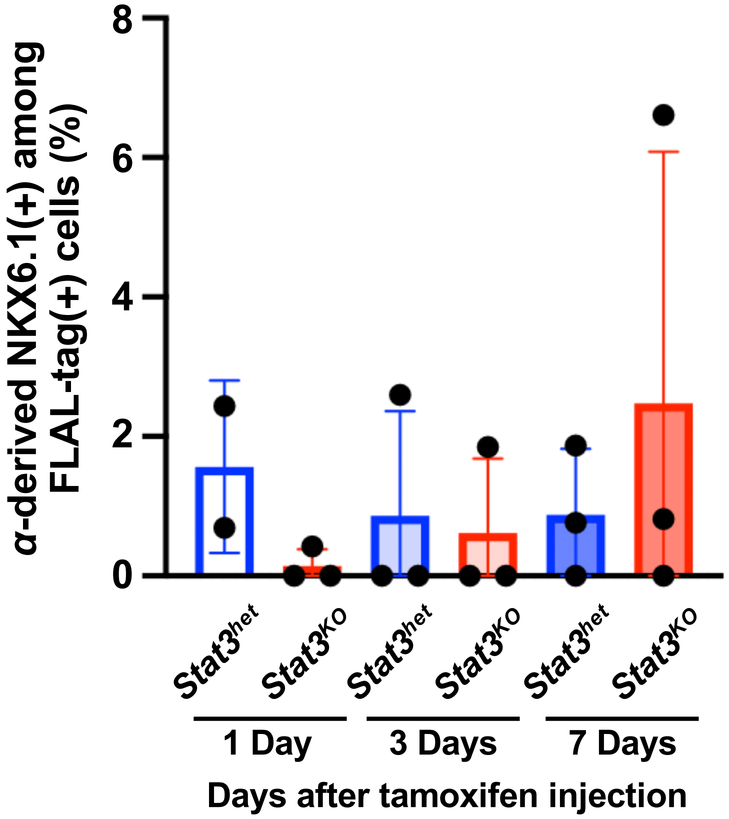

Figure S6. NKX6.1 expression in α-cell-derived PDX1-expressing cells.
The percentage of NKX6.1-expressing cells among FLAG-tag-positive cells expressing ectopic PDX1 in *αPdx1; Stat3*^{Hetero} and *αPdx1; Stat3*^{KO} mice (n = 3–5 in each group).

Figure S7

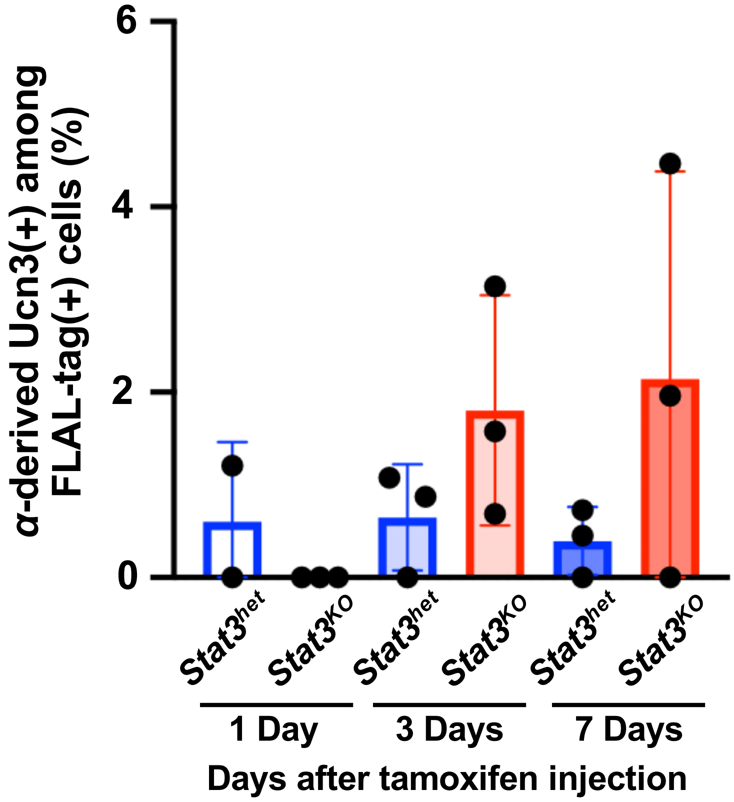

Figure S7. UCN3 expression in α-cell-derived PDX1-expressing cells.
The percentage of UCN3-expressing cells among FLAG-tag-positive cells, expressing ectopic PDX1, was counted in *αPdx1; Stat3*^{Hetero} and *αPdx1; Stat3*^{KO} mice (n = 3–5 in each group).

Figure S8

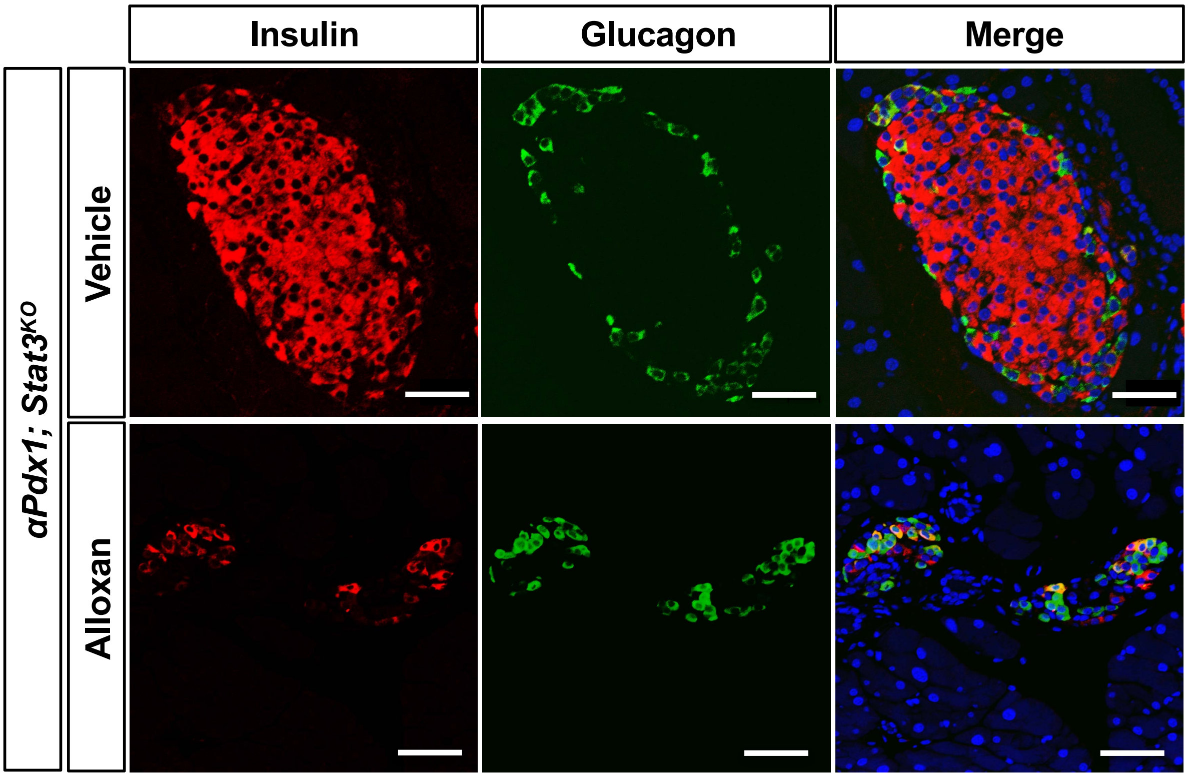

Figure S8. Representative images of alloxan-treated islets of *αPdx1; Stat3^{KO}* mice. Immunostaining against insulin (red) and glucagon (white) was performed in the pancreas of *αPdx1; Stat3^{KO}* mice after the administration of alloxan or vehicle. Scale bars, 50 µm.

Figure S9

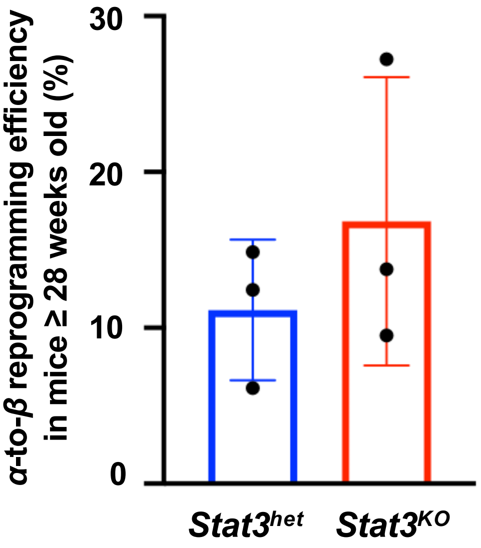

Figure S9. α-to-β reprogramming efficiency in aged mice.
The percentage of reprogrammed β cells among FLAG-tag-positive cells was counted in *αPdx1; Stat3*^{Hetero} and *αPdx1; Stat3*^{KO} mice at the age of 28 weeks or older (n = 3 in each group).

Figure S10

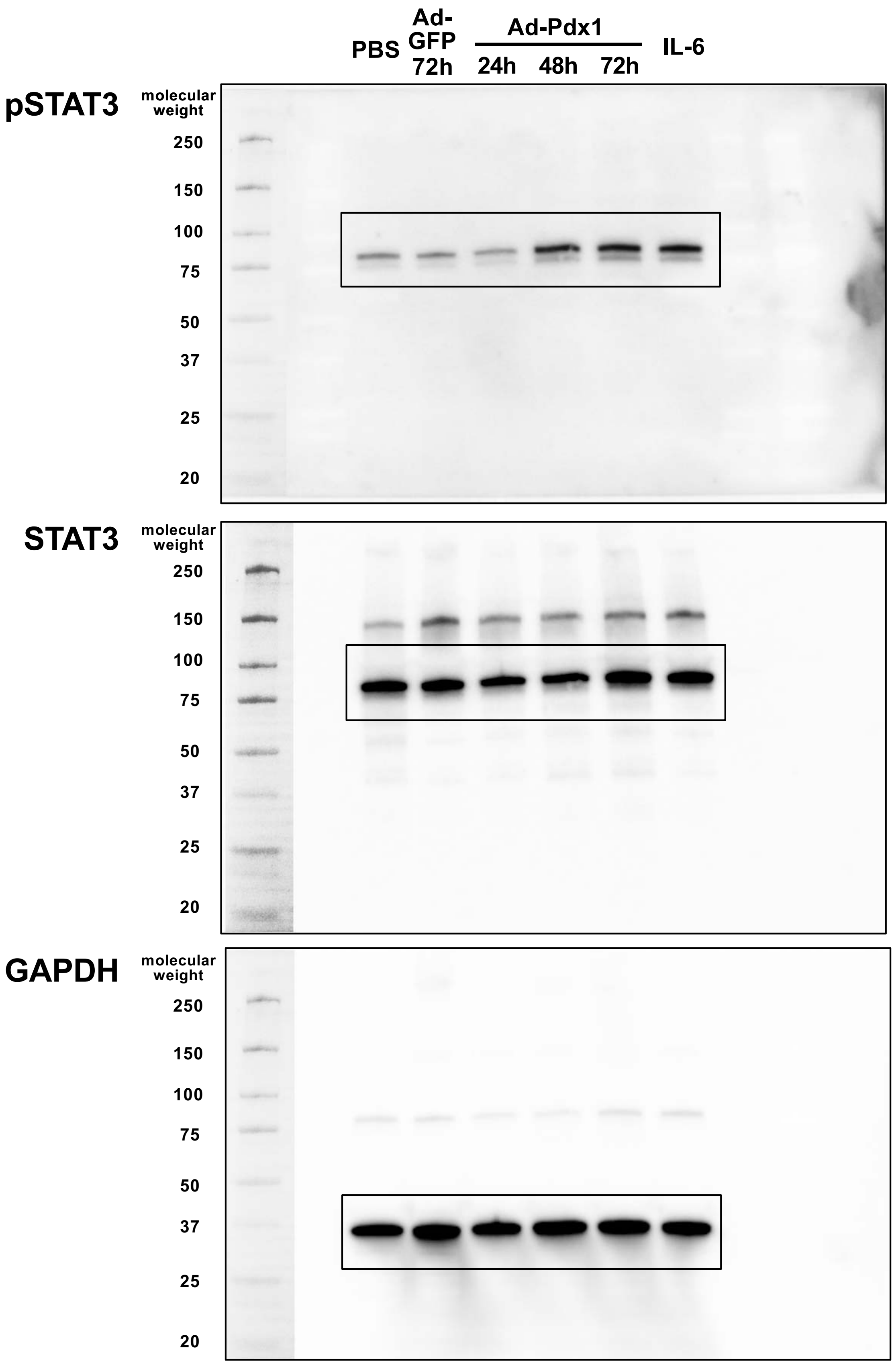

Figure S10. Uncropped images of key panels in main figures.
Black boxes indicate the cropped portion of each immunoblot presented in Figure 1.
